# Sulforaphane from Cruciferous Vegetables: Recent Advances to Improve Glioblastoma Treatment

**DOI:** 10.3390/nu10111755

**Published:** 2018-11-14

**Authors:** Giulia Sita, Patrizia Hrelia, Agnese Graziosi, Fabiana Morroni

**Affiliations:** Department of Pharmacy and BioTechnology, Alma Mater Studiorum—University of Bologna, via Irnerio 48, 40126 Bologna, Italy; giulia.sita2@unibo.it (G.S.); agnese.graziosi2@unibo.it (A.G.); fabiana.morroni@unibo.it (F.M.)

**Keywords:** sulforaphane, glioblastoma multiforme, apoptosis, chemoprevention

## Abstract

Sulforaphane (SFN), an isothiocyanate (ITC) derived from cruciferous vegetables, particularly broccoli and broccoli sprouts, has been widely investigated due to its promising health-promoting properties in disease, and low toxicity in normal tissue. Although not yet fully understood, many mechanisms of anticancer activity at each step of cancer development have been attributed to this ITC. Given the promising data available regarding SFN, this review aimed to provide an overview on the potential activities of SFN related to the cellular mechanisms involved in glioblastoma (GBM) progression. GBM is the most frequent malignant brain tumor among adults and is currently an incurable disease due mostly to its highly invasive phenotype, and the poor efficacy of the available therapies. Despite all efforts, the median overall survival of GBM patients remains approximately 1.5 years under therapy. Therefore, there is an urgent need to provide support for translating the progress in understanding the molecular background of GBM into more complex, but promising therapeutic strategies, in which SFN may find a leading role.

## 1. Introduction

Primary Central Nervous System (CNS) tumors refer to a variety of tumors arising from cells within the brain, and among them, glioblastoma multiforme (GBM) is one of the most aggressive and malignant forms [1]. Primary brain tumors account for about 2% of all cancers, with an overall annual incidence of 24.8 per 100,000 population [2]. The incidence varies with advancing age, sex, and ethnic origin [1]. The peculiar nature and locations of CNS tumors usually mean that the treatments available, including surgery, radiotherapy, or chemotherapy, are not successful in eradicating all the tumor cells.

**Figure 1:**
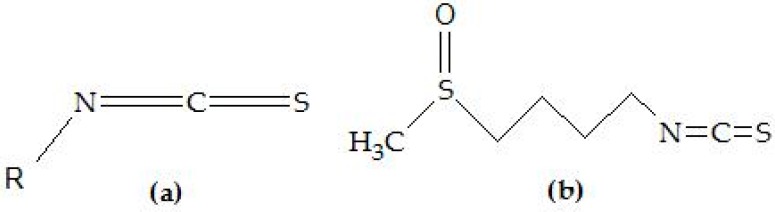
Chemical structures of (**a**) isothiocyanates (ITCs) and (**b**) sulforaphane (SFN).

The limited success of the available therapies has pushed the research towards therapeutic strategies based on chemoprevention. The term chemoprevention refers to the use of agents able to prevent, block, or even reverse the process of tumor development before clinical manifestation of the disease [3]. The main purpose of chemoprevention is to delay the onset of cancer and to decrease its incidence. Therefore, all these strategies require the use of compounds that inhibit specific molecular steps in the carcinogenic pathway. Bioactive phytochemicals have shown promising therapeutic effects on brain cancers and other forms of cancer [4,5,6,7], as well as neuroprotective effects on Alzheimer’s disease, Parkinson’s disease, and other neurodegenerative diseases [8,9,10]. Assessing the real efficacy and bioavailability of these compounds currently represents a matter of great debate. In particular, there is a growing interest in identifying specific chemoprotective constituents in cruciferous vegetables and their mechanisms of action. The isothiocyanate (ITC, Figure 1a) sulforaphane (SFN, Figure 1b), which is converted from a major glucosinolate in broccoli/broccoli sprouts, has been shown to prevent chemically induced cancers in animal models and to inhibit the growth of established tumors [11,12,13].

Here, we provide an overview of the research from 2006 to 2018 by the use of PubMed (https://www.ncbi.nlm.nih.gov/pubmed/) regarding the ability of SFN in targeting GBM, including the mechanisms of action involved in the benefits it provides.

### Glioblastoma: Pathophysiology and Therapies

GBM is a malignant tumor originating from glial cells. According to the 2016 World Health Organization (WHO) classification, GBM is a grade IV astrocytoma [14], which means it is a rapidly growing and highly malignant tumor. GBMs can be “primary” or “secondary” depending if they arise de novo or if they evolve by progression from a lower-grade glioma [15]. Notably, the vast majority of GBMs (~90%) develop rapidly de novo in elderly patients [16]. GBM may present itself with headaches, seizures, or focal neurological symptoms. Due to its aggressive nature, symptoms may develop rapidly. In spite of all efforts, the estimated overall survival of GBM patients is less than 1.5 years under therapy, and the five-year survival rate is 5% [17]. The poor survival is partly attributable to the nature of the tumor itself: GBM is generally characterized by rapid cell proliferation and insufficient vascularization that lead to the formation of hypoxic tumor areas [18]. These extremely low oxygen levels could generate necrotic zones surrounded by the so-called pseudopalisading GBM cells, which are densely packed hypoxic tumor cells. It is known that these cells express hypoxia-regulated genes that modulate key processes associated with tumor aggressiveness [19]. Hypoxia is also a well-recognized feature of the tumor microenvironment and has been related to fatal outcome and resistance to therapies in different tumors [20]. Up-regulation of vascular endothelial growth factor (VEGF) from intratumoral hypoxia and dysregulation of growth factor signaling play crucial roles in the pathophysiology of tumor resistance and recurrence [21]. Thus, the molecular biology underlying GBM is complex and not fully understood, which highlights the urgent need of specific treatment strategies.

The most effective available therapies for GBM, especially in elderly patients, include surgical resection, adjuvant radiotherapy and chemotherapy with temozolomide (TMZ) [22]. TMZ is an alkylating agent and its main mechanism of action is the arrest of the cell cycle at G2/M checkpoint, leading to apoptosis of GBM cells [23]. Unfortunately, there are still many unsolved problems in the treatment of GBM. First of all, the complete resection of gliomas is almost impossible, and due to their infiltrative nature the tumor recurs, leading to patients’ death. Second, the efficacy of chemotherapy is further reduced by the blood brain barrier (BBB) that limits the delivery of drugs into the brain [24]. Moreover, TMZ is not always effective [25]. The critical problem to solve in chemotherapy is the cancer drug resistance that is controlled by different intrinsic and extrinsic factors, such as the tumor microenvironment, the potency of the anticancer drugs, the heterogeneity of cancer cells, and the response of cancer cells to the drugs [26]. Thus, there is an urgent need for novel, targeted, and effective therapies for GBM.

## 2. Sulforaphane in Cancer

Numerous studies have identified different natural products and their analogues as potential chemopreventive agents [27,28,29,30]. In the last decade, cancer research area has developed a consistent interest in diet-derived chemopreventive agents. The application of phytochemicals represents a very promising and modern strategy for cancer prevention and treatment [31]. Moreover, several reports have proposed that some phytochemicals may act as sensitizers, leading to the increased effectiveness of conventional radiotherapy [32,33]. ITCs are secondary plant metabolites that are found in high concentration in cruciferous vegetables, as a system of defense against pathogen attack, and they arise from the hydrolysis of glucosinolates by the enzyme myrosinase. They are a family of small organosulfur molecules characterized by the presence of an –N=C=S group with antioxidant and anticancer properties. The formation of ITCs depends on a broad spectrum of plant intrinsic factors, such as the glucosinolates’ concentration and the myrosinase’s activity, as well as on several extrinsic factors, such as the post-harvesting processes, mastication, and digestion [34]. The great variability in the formation of ITCs and other breakdown products is determined by all these factors [34]. Nowadays, ITCs have gained an increasing research interest. As suggested by epidemiological studies dietary intake of cruciferous vegetables (broccoli, cauliflowers, and Brussels sprouts) is negatively related to cancer risk, such as breast, stomach, prostate, bladder and lung cancers, and this effect is demonstrated to result from the ITCs activity in such vegetables [35].

Among ITCs, SFN, 1-isothiocyanato-4-(methylsulfinyl)-butane, is the most extensively studied [28,36,37]. The bioavailability and metabolism of ITCs are key issues when considering the potential impact of these compounds on human health. SFN is conjugated in vivo with glutathione (GSH) to produce SFN-GSH, SFN cysteine glycine (SFN-CG), SFN-cysteine (SFN-Cys), and SFN-N-acetylcysteine (SFN-NAC) via the mercapturic acid pathway [38,39,40]. Different pharmacokinetic studies have already shown that SFN is well distributed in the body and it can reach target tissues in the active form [41,42]. SFN shows its chemoprotective and chemotherapeutic properties through its pleiotropic activity by modulating different mechanisms involved in the pathogenesis of cancer. This ITC is considered to be a phytochemical with low toxicity. Interestingly, Socala et al. [43] evaluated some preliminary toxicity data of SFN in mice after intraperitoneal (i.p.) administration. Their results showed that SFN at high doses (250–300 mg/kg) produced significant sedation, decreased muscle strength, and impaired motor performance. Based on the results from toxicity studies, the TD50 and LD50 resulted in 191.58 mg/kg and 212.67 mg/kg, respectively. Moreover, several clinical trials proved the safety and tolerance of SFN [44,45,46].

Numerous studies have demonstrated multiple mechanisms by which SFN may exert its effects, as the inhibition of procarcinogen metabolism, the alteration of the phase 3 metabolism, the induction of apoptosis and inhibition of cell cycle progression, and the modulation of oxidative stress [47,48,49,50]. The proapoptotic activity of SFN is mediated by complex and diversified mechanisms of action. Apoptosis may be regulated by the alteration of tubulin polymerization [51], increased oxidative stress [52] and decreased intracellular antioxidant levels [53]. Cellular reactive oxygen species (ROS) generation by SFN plays a crucial role in the initiation of the apoptotic death mediated by this ITC [54]. However, it is important to underline that ROS generation by SFN is specific for tumor cells, because normal cells are resistant to its proapoptotic effects [55]. As many studies have shown, SFN induces apoptosis in many different cell types, as in prostate cancer, where the ITC is able to activate caspases, to decrease DNA content and to increase Bax:Bcl-2 ratio [56]. Following SFN treatment, the activation of caspases was also demonstrated in medulloblastoma and human pancreatic cancer cells [53,57]. Finally, in GBM cells, the apoptosis induced by SFN seems to be driven by both caspase-dependent and -independent apoptotic pathways [56]. Beside the ability to promote apoptosis in different cells types, SFN treatment additionally showed the capacity to arrest the cell cycle in the G2/M phase and to inhibit the proliferation in colon cancer cells [58,59]. In the last years, several studies showed the effect of SFN on tumor cell migration and invasion. Interestingly, SFN counteracted migration in prostate cancer, invasion in breast cancer, and decreased both migration and invasion in bladder cancer and oral carcinoma [60,61,62,63]. Furthermore, SFN suppressed azoxymethane-induced colonic aberrant crypt foci (ACF) [64] and prevented polyps in adenomatous polyposis coli (Apc)/multiple intestinal neoplasia (Min) mice [65]. Jackson et al. recorded a significant decrease in tumor mass and an increase in apoptotic cascade, after implanting murine mammary carcinoma cells in BALB/c mice and subsequently treated with SFN for 13 days [51]. In addition, Singh et al. demonstrated that oral administration of SFN significantly inhibited the growth of prostate cancer cell xenografts in nude mice, and increased the expression of proapoptotic proteins, such as Bax and Bid [66].

Multiple mechanisms are involved in the anticancer activity of SFN, including the activation of detoxification enzymes, the induction of oxidative stress, the checkpoint activation in DNA damage, and the inhibition of histone deacetylase (HDAC) on histone H3 and H4 promoters of genes, such as p21 [67,68]. Previous studies on different tumors highlighted that several survival signaling pathways could be modulated by SFN. For example, SFN was able to activate phase 2 antioxidant enzymes via the Kelch-like ECH-associated protein 1 nuclear factor E2-related factor 2 (Keap1/Nrf2) [69,70,71,72] and significantly decrease the expression of phosphorylated c-Jun N-terminal kinase (p-JNK), phosphorylated extracellular signal-regulated kinases (p-ERK), protein kinase B (p-Akt) and β-catenin, and then interrupt the mitogen-activated protein kinase (MAPK), phosphatidylinositol-4,5-bisphosphate 3-kinase (PI3K)/Akt and Wnt signaling pathways [73,74].

## 3. Sulforaphane in Glioblastoma

The potential application of phytochemicals in the treatment of human tumors has been investigated for a long time. The growing interest of the scientific community lies in the fact that these compounds have been recognized as safe agents. Because of the relative low toxicity to normal cells and general availability of SFN, it has been extensively studied for its anti-cancer activities [75]. Moreover, SFN can rapidly cross the BBB and accumulate in the CNS after i.p. administration [76,77]. In recent years, a growing interest has also been directed towards application of SFN in GBM to induce apoptosis, and to inhibit both growth and invasion of GBM cells [78]. Furthermore, SFN can overcome the chemoresistance of tumor cells [79,80].

Therefore, the use of a pleiotropic agent that may affect specific cancer cell features could be a successful strategy to fight GBM [81]. Here, we would like to summarize the effects of SFN focusing on pro-apoptotic, anti-invasion, anti-proliferative, and anti-chemo/radioresistance activities of this ITC in GBM treatment.

### 3.1. Sulforaphane and Blood Brain Barrier

The BBB is a complex cellular vascular structure that prevents the penetration of macromolecules and small molecules into the CNS, counting anticancer drugs, which are not able to reach the tumor mass [82]. With its extremely selective permeability, the BBB can be considered a key factor in the poor distribution of potentially effective therapeutic agents in CNS disorders [83]. For this reason, new strategies for a more efficient drug delivery across the BBB are urgently needed. Several studies have already been focalized on the enhancement of the permeability of the BBB to improve therapeutic outcomes [84].

The BBB is composed primarily by neurovascular units (NUs) that comprises endothelial cells supported by the neuroglia [85]. Beside the NUs, the selectivity of the BBB is additionally regulated by numerous endothelial tight junctions (TJs), and by the presence of ATP-binding cassette (ABC) transporters, such as P-glycoprotein, which can increase drug influx to the CNS [86]. This physiological architecture makes the BBB poorly permeable to most of the phytochemical compounds and their related metabolites. Despite extensive research in understanding the function and activity of SFN, little is known regarding the tissue distribution of SFN and its metabolites. Clarke et al. [39] demonstrated that, following SFN gavage, its metabolites were detected in all mice tissues at 2 and 6 h. In particular, the highest concentration in the brain was reached at 2 h. Although the ability of SFN metabolites to cross the BBB is poorly known, in the same study they reported low concentration of these metabolites in the brain, which likely indicates that they can cross the BBB, though not readily [39].

The integrity of the BBB changes during the development of GBM [87]. Generally, it is possible to observe abnormal structural variations in endothelial TJs that lead to an enhanced permeability of the BBB when compared with healthy tissue [88,89]. It is important to highlight that GBM also represents the most vascularized tumors in humans [82]. The growth of this glioma is very rapid and associated with the formation of new vessels, and their density is an important indicator of the prognosis in patients [90]. Although the inhibition of tumor angiogenesis may be a promising strategy for the treatment of GBM [91], the fact that cancer cells infiltrate diffusely without necessarily requiring angiogenesis, indicates that the invasion is also associated with pre-existing blood vessels [92]. Vessels involved in the growth of GBM do not respond to vasoregulatory factors released by astrocyte, resulting in the loss of the TJ’s and BBB’s integrity [93]. The disruption of BBB integrity is responsible for empowering the invasion of tumor cells, however, at the same time, it may be helpful for a better drug delivery into tumor cells [89]. In summary, BBB maintain its properties in the periphery of GBM, resulting in the failure of the current therapy, and at the same time, it loses integrity to allow the tumor infiltration [84].

During tumor progression, the expression of matrix metalloproteinases (MMP), in particular MMP-9, significantly increases and it is associated with the alteration of BBB [94]. MMP-9 has already demonstrated to play a crucial role in the structural organization of endothelial cells [95]. Notably, Annabi et al. showed that the increased secretion of MMP-9 by human brain microvascular endothelial cells was decreased by SFN treatment. Moreover, SFN reduced cells migration, showing a potential role for this ITC to inhibit the functions mediated by MMP-9 in GBM [96]. There is no controversy about the significance of improving drug delivery across the BBB. In this view, the attractive modulation of BBB for therapeutic benefit might be an interesting mechanism behind the chemopreventive activity of SFN [36].

### 3.2. Anti-Proliferation and Apoptosis

Many studies have reported that SFN has cytotoxic and proapoptotic activities in different types of cancer [97,98,99,100]. Interestingly, SFN has a modest effect on healthy brain cells and does not affect normal human mesenchymal stromal cells at concentrations where cancer cells will die off [81]. Like two sides of the same coin, every defect or abnormality in the apoptotic machinery may also be a potential target of cancer treatment. Any strategy that can restore the apoptotic pathways towards normality have the potential to eradicate cancer cells, which stay alive thanks to these defects.

Induction of apoptosis associated with increased intracellular calcium concentration (Ca^2+^) has been demonstrated in various in vitro models [101,102]. An increase of intracellular Ca^2+^ can trigger downstream adverse events including mitochondrial dysfunction, up-regulation of calpain, and cytochrome c release for the activation of a caspase cascade, leading to cytoskeletal damage and apoptosis. Karmakar et al. demonstrated in two different GBM cell lines that SFN caused endoplasmatic reticulum (ER) stress to raise Ca^2+^ and release caspase-12. Once activated by calpain, caspase-12 leads to caspase-9 activation. Moreover, SFN mediated both caspase-dependent apoptosis by increasing the Bax:Bcl-2 ratio and mitochondrial release of several pro-apoptotic molecules, such as cytochrome c and second mitochondria-derived activator of caspase/direct inhibitor of apoptosis-binding protein with low pI (Smac/Diablo), and caspase-independent apoptosis by the apoptosis-inducing factor (AIF) [56,78]. These effects of SFN on GBM cells are further confirmed by Miao et al. [103]. They also demonstrated that SFN induces apoptosis of GBM cells increasing ROS levels in these cells. However, other reports revealed that SFN may protect normal cells against oxidative stress [104,105]. These paradoxical SFN activities are related to the intrinsic high level of ROS in cancer cells, which might contribute to amplify the death signal induced by anti-cancer agents. In contrast, this does not happen in normal cells, in which the same increase of the ROS level evokes a cytoprotective effect [106]. Interestingly, SFN-generated ROS in GBM cells are formed at the mitochondrial respiratory chain level [81]; rotenone or myxothiazol (mitochondrial respiratory chain Complex I and III inhibitors, respectively) abolished ROS formation in Jurkat leukemia cells [107]. It is possible that SFN-induced mitochondrial ROS might trigger DNA damage and consequent apoptosis, as demonstrated by the increased single-strand breaks after SFN treatment in GBM cells [81]. Studies have shown that the signal transducer and activator of transcription 3 (STAT3) mediates proliferative signals and it is constitutively activated in GBM [108,109]. Different studies demonstrated that SFN treatment could induce the time- and dose-dependent down-regulation of Janus kinase 2 (JAK2) and Src kinases phosphorylation in GBM cells via post-translational modification of cysteine residues, which can potentially inhibit the STAT3 pathway in a ROS-dependent manner [103,110]. Even more interestingly, the activation and interaction between STAT3 and the nuclear factor kappa-light-chain-enhancer of activated B cells (NF-κB) play crucial roles in controlling the dialog between cancer cells and their microenvironment, especially with immune cells that infiltrate tumors. NF-κB and STAT3 are strictly involved in the control of apoptosis-based tumor-surveillance, tumor angiogenesis and invasiveness [111]. Additionally, SFN treatment caused down-regulation of NF-κB in human GBM cells [56,112]. In particular, the mechanisms of action of SFN to prevent GBM cell survival signals include both the inhibition of two inhibitor-of-apoptosis proteins (IAPs), and the up-regulation of IκBα, an endogenous inhibitor of NF-κB [56].

Another issue in treating GBM is the presence of GBM stem cells (GSCs) that are extremely resistant to therapy and critical for tumor invasiveness [113]. GSCs are manly localized in the perinecrotic hypoxic area, and CD133 and SOX2 are largely used as cancer stem cells markers. Interestingly, Bijangi-Vishehsaraei et al. showed that SFN induced apoptosis in CD133-positive GSCs and significantly inhibited the survival of the CD133-positive and SOX2-expressing GBM spheroids obtained from GBM cell lines [81]. These effects are probably triggered by the up-regulation of hypoxia-inducible factor (HIF)-1α, the master transcriptional regulator of cellular response to hypoxia, and of the hypoxia-mediated maintenance of GSCs [114].

It is interesting to highlight that SFN is able to inhibit significantly tumor growth in cancer xenografts, i.e., severe combined immunodeficiency (SCID) mice inoculated with GBM8401 cells [112] and NOD scid gamma mice (NSG) inoculated with early-passage human GBM10 primary cultures [81]. Oral SFN administration (100 mg/kg/day) delayed the tumor’s growth and enhanced cell death of ectopic GBM10 xenografts. Moreover, histological analyses of mouse tissues showed no cytotoxicity in the liver, lung, brain, spleen, and kidney [81].

We have already discussed that in vivo SFN is metabolized to produce different metabolites; among them, SFN-Cys has gained increasing attention of researchers. In vivo SFN-Cys has a longer half-life and retention time, and inhibits the HDAC more efficiently, which is strongly related to cell growth, as compared to SFN alone [39,115]. A recent in vitro research conducted by Wu et al. showed that the treatment with different doses of SFN-Cys (up to 45 μM) for 24 h induced cell apoptosis dose-dependently by up-regulating Bax:Bcl-2 ratio, and subsequently, caused the loss of MMP through the activation of ERK1/2 pathway [38]. Furthermore, in the same study, the authors demonstrated that the programmed cell death induced by SFN-Cys was activated by the release of Smac, and then by the neutralization of the IAP proteins [38]. The authors suggested that the pro-apoptotic potential of this metabolite is not only related to the extrinsic apoptotic pathway, but also to the intrinsic apoptotic pathway and the ER stress-mediated pathway. Table 1 summarizes the principal studies focalized on the anti-proliferative and the pro-apoptotic role of SFN in GBM.

### 3.3. Anti-Invasion and Anti-Migration

The poor prognosis of GBM primarily originates from its highly invasive potential and by its rapid growth profile [117,118,119]. Thus, an efficient therapeutic strategy should be able to inhibit both growth and invasion of tumor cells [120].

As already known, SFN could inhibit migration and/or invasion in many kinds of cancer cells [60,61,62]. In 2016, Zhang et al. investigated the effects of SFN on U251MG GBM cells to assess the potential effectiveness of this ITC to counteract the tumor growth and its infiltrative potential. To this aim, authors treated U251MG cells with SFN (up to 40 μM) for 24 h to investigate its anti-invasion activity. The results obtained showed that SFN treatment reduced the invasive potential of GBM cells in a dose-dependent manner [78], as already demonstrated in different cancer cell lines [61,63]. Furthermore, several studies have shown that SFN may be responsible for the activation of ERK1/2 and the consequent induction of apoptosis in human brain glioma and neuroblastoma cells [112,121]. In 2013, Li et al. demonstrated that transient activation of ERK1/2 can contribute to GBM migration and invasion [122,123]. Tumor cell invasion through the basement membrane is an essential step for the propagation of cells from the primary site to distal secondary sites. In this process, MMPs play a central role, because they might damage basement membrane to create space for GBM cells and promote the invasion cascade. Increased expression of MMP-2 occurs in different human tumors, including breast, ovarian, prostate, and melanoma [124]. Additionally, human GBM samples express high levels of MMP-2 and MMP-9 as compared to normal brain tissues, and these levels increased with tumor progression [125,126,127]. Coniglio and Segall demonstrated that the invasiveness of GBM cells was significantly decreased by the inhibition of MMPs [128]. Notably, several studies showed that SFN down-regulated MMP-2 expression in different tumor cell lines, modulating cellular survival pathways, such as ERK1/2 [62,63,123,129,130,131]. Moreover, Galectin-3 and E-cadherin, cell actors involved in cancer invasion, are highly expressed in GBM and are modulated by MMPs [132]. The results reported by Zhang et al. have shown that SFN treatment increased the protein levels of E-cadherin and decreased Galectin-3, MMP-2 and MMP-9 [78]. The protein kinase ERK1/2 modulates the expression of CD44 glycoprotein, an adhesion molecule involved in tumor cell migration and invasion. Interestingly, Li et al. demonstrated that the treatment of U87MG and U373MG cells with SFN up to 30 μM for 24 h inhibited the invasive potential of these cells through ERK1/2 activation, or possibly preventing the nuclear dephosphorylation of this kinase [47]. Consequently, ERK1/2 controls the expression and activity of MMP-2, as well as invasion. Moreover, SFN seems to be able to reduce morphological changes involved in cell adhesion, migration, invasion and the entire process of metastasis in U87MG and U373MG cells [133,134]. In summary, SFN may inhibit cell invasion via ERK1/2 signaling pathway [47], and modulate MMP-2 and MMP-9 expressions [38]. Furthermore, SFN has shown the ability to regulate Galectin-3 and E-cadherin not only in GBM, but also in different tumor cell lines [38,135,136]. The following Table 2 summarizes the principal studies focalized on the anti-migration role of SFN in GBM.

### 3.4. Anti-Chemo/Radioresistance

An unsuccessful therapeutic outcome in GBM is often related to the development of chemotherapy resistance. Promising results suggest that combining chemopreventive agents with chemotherapy or radiotherapy may not only enhance antitumor activity, but also reverse drug resistance and make cancer cells more susceptible to chemotherapeutic drugs [137,138].

As we already mentioned, the first-line agent in the treatment of GBM is TMZ, which triggers cell death through the formation of O-6-methylguanine [139,140]. Many studies have been focalized on improving TMZ efficacy to increase the overall survival of GBM patients.

The therapeutic resistance to TMZ can occur at different levels, and it may be related to the enzyme O-6-methylguanine-DNA methyltransferase (MGMT), whose expression varies widely in different kinds of tumor cells [141]. Increasing evidences suggest that MGMT overexpression is able to counteract TMZ-induced cell death; moreover, patients with high MGMT expression have a poorer prognosis as compared to those with low expression [142]. Importantly, MGMT promoter methylation appears to be a predictive biomarker associated with improved clinical outcomes and survival [143,144].

An oncogenic role for activated NF-κB has been highlighted in a variety of tumors to promote cell proliferation and invasion, to induce angiogenesis and metastasis, and to prevent apoptosis [145]. Many chemotherapeutic agents and radiation may induce NF-κB activity in different cancer cells, which is mainly related to drug resistance [146], because of its involvement in MGMT transcription [145,147]. Therefore, inhibiting the NF-κB-MGMT pathway may represent an efficient strategy to overcome TMZ-resistance, increasing sensitivity of GBM cells to alkylating chemotherapeutic treatment and may help in overcoming chemoresistance induced by the treatment.

A novel approach to GBM therapy is the combination of natural compounds with TMZ [54]. In this view, Lan et al. evaluated the activity of SFN in sensitizing different malignant glioma cell lines resistant to TMZ treatment. The study showed that SFN reversed TMZ-chemoresistence in GBM cells by the down-regulation of MGMT expression via NF-κB signaling pathway [116].

Most of the studies on the possible sensitizing efficacy of SFN have been focused on the interaction of this ITC with the tumor necrosis factor (TNF)-related apoptosis-inducing ligand (TRAIL). TRAIL is the natural ligand for apoptotic receptors that contributes to TMZ resistance and triggers apoptosis in different in vitro and in vivo cancer models, without conferring significant toxicity to normal cells [41,148,149]. Although TRAIL exerts promising anticancer effects, several primary tumors, such as GBMs, present a phenotype quite resistant to apoptosis induced by TRAIL [150,151]. Interestingly, SFN is able to sensitize different TRAIL-resistant human cancer lines to TRAIL-induced apoptosis, mainly by triggering death receptors [152,153]. Moreover, in 2012, Kaminski et al. demonstrated that SFN in combination with exogenous TRAIL could also induce endogenous TRAIL expression in colorectal cancer cells [154]. Notably, TMZ might act as a TRAIL “sensitizer”, defeating resistance by up-regulating the expression of death receptors, leading to the activation of caspases [155]. Moreover, TRAIL and TMZ may share other mechanisms of resistance, like up-regulation of anti-apoptotic IAP proteins and down-regulation of pro-apoptotic Bcl-2 proteins [156,157].

In an another study, Lan et al. investigated the potential activity of SFN treatment as a repressor of the Wnt/b-catenin signaling pathway, involved in survival processes [79]. Several studies suggest that microRNAs (MiRNAs) may be considered to be promising diagnostic biomarkers and therapeutic targets in different cancers, including gliomas [158]. Among them, miR-21 plays a critical role in several aspects of carcinogenesis as cellular proliferation and migration that are regulated by the atypical activation of the Wnt/b-catenin pathway [159]. The authors demonstrated that the therapeutic efficacy of TMZ can be enhanced by targeting miR-21 expression. As reported, the up-regulation of miR-21 is related with poor prognosis in GBM [160]. Indeed, miR-21 takes part in TMZ-chemoresistance by decreasing Bax:Bcl-2 ratio and caspase 3 activity [161]. Interestingly, SFN treatment increases caspase 3/7 activity and Bax:Bcl-2 ratio, and promotes TMZ-induced apoptosis in GBM cells by down-regulating miR-21 expression through Wnt/b-catenin signaling [79]. Table 3 summarizes the principal studies that focalized on the anti-chemo/radioresistance induced by SFN in GBM.

## 4. Conclusions and Future Directions

**Figure 2:**
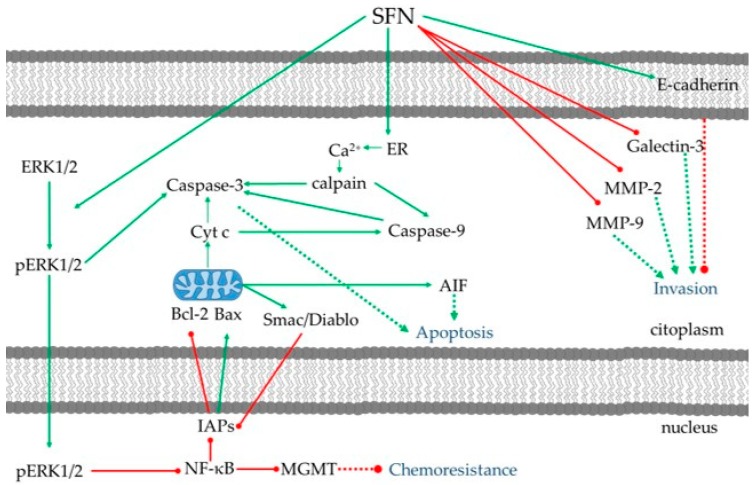
Summary of the multiple signaling pathways of SFN in glioblastoma (GBM). SFN may induce apoptosis through endoplasmatic reticulum (ER) stress, or through the inhibition of survival signals and promotion of pro-apoptotic molecules. SFN is also able to reduce tumor invasiveness and to counteract chemoresistance.

To date, it has not yet been possible to discover effective therapies for GBM, though there are several attempts to improve the unfavorable patient outcomes. Moreover, the current chemotherapy may lead to drug resistance in GBM treatment, as it severely destabilizes the cell metabolism and cell signaling network. Here, we have reviewed a number of studies that report the potential role of SFN as new alternative to complement preexisting treatments. Several of the studies reviewed emphasize the potent anti-GBM activity of SFN that targets apoptosis and cell survival pathways and also show a remarkable selectivity of action against tumor cells (Figure 2). Taken together, the results of these studies support further investigations using SFN in animal models of GBM.

Finally, if the expected results will be confirmed, the antitumor activities ascribed to SFN could be investigated in humans. In this view, prospective randomized clinical trials should be done to explore the use of adjunctive SFN therapy in better targeting resistance and synergistically improving upon standard treatments.

## Figures and Tables

**Table 1:** Summary of studies focused on the anti-proliferative and pro-apoptotic properties of sulforaphane (SFN) in glioblastoma (GBM).

Model	Dose	References
U251	10–40 μM	[78]
T98G	20–40 μM	[56]
U87		
GBM 8401	12.5–50 μM	[112]
U252	10–40 μM	[103]
U87		
U87	5–30 μM	[81]
M-HBT-32		
U373	30–70 μM	
U118		
SF767		
U87 spheroids		
GBM43 spheroids	10–50 μM	
M-HBT-161 spheroids		
NSG ^1^ mice implanted with GBM10 cells	100 mg/Kg/day per os	
T98G	10–30 μM	[116]
U87-R		
U373-R		

^1^ NSG: NOD severe combined immunodeficiency gamma mice

**Table 2:** Summary of studies focused on the anti-migration properties of SFN in GBM.

Model	Dose	References
U87	10–30 μM	[47]
U373		
U251	10–40 μM	[78]
GBM 8401	2.5–10 μM	[112]
T98G	10–30 μM	[116]
U87-R		
U373-R		

**Table 3:** Summary of main studies focused on the anti-chemo/radio resistance induced by SFN in GBM.

Model	Dose	References
LN229	5–40 μM	[79]
U251		
T98G	10–30 μM	[116]
U87-R		
U373-R		
Nude mice implanted with U373-R	50 mg/Kg/day i.p.	
